# Non-Vital Teeth

**Published:** 1919-06

**Authors:** Arthur H. Merritt

**Affiliations:** 59 West 46th Street, New York, City


### Non-Vital Teeth; Their Etiology, Pathology and Treatment.

Arthur H. Merritt, D. D. S., 59 West 46th Street, New York, City.

One of the most prolific sources of metastatic infections is the non-vital or so called "dead tooth." Of all chronic mouth infections it is the most common. No age is exempt. It is the frequency with which they occur, and the large number which may exist in a given mouth, which gives them their importance. Their relationship to such diseases as arthritis, myositis, neuritis, endocarditis, anemia, etc., has been repeatedly demonstrated. That such relationship actually exists in a given case which may present itself for treatment is of course never certain. The only safe rule is to remove such foci inasmuch as they are always potential if not actual causes of disease. No physical examination is complete which does not include a careful examination of all teeth, clinical and roentgenographic. Reliance cannot be placed upon subject symptoms. As a rule, chronic infections of this kind are without local symptoms. Nor can one depend upon the most painstaking clinical examination alone, though such examination should be made to determine the number and location of such non-vital teeth as may be present. Roentgenograms should always be made of every tooth, for in no other way can its exact status be determined. It should be remembered that periapical infection, or so called abcesses, take place only on non-vital teeth, never on teeth with healthy pulps. There is therefore no necessity of making a roentgenographic examination of the entire mouth, providing a careful clinical examination has been made and the presence or absence of non-vital teeth established.

### Etiology.

Chief among the causes of non-vital teeth are dental caries, pulp infection (usually the result of caries) thermal shock, trauma and drugs used for devitalization and pulp removal. The most common of these is caries. Once this has developed in a tooth it is only a question of time unless arrested, before it will cause its devitalization. The nearer it approaches the pulp the greater the danger from this cause,—the chief argument for frequent examination and cleaning of the teeth, thereby limiting as far as possible the development and progress of dental caries. Thermal shock, through the influence of temperature changes may cause death of the tooth pulp. Teeth with large fillings in them, or with gums receded exposing portions of the root, are especially prone to thermal shock. Trauma, often too slight to cause fracture, may so interfere with the circulation of the pulp as to cause its death. Dental caries is however chief among the causes of non-vital teeth. Not until this has been prevented will the problem of non-vital teeth be solved.

### Pathology.

The pathology of the non-vital tooth first finds expression in the pulp itself, usually in the form of hyperemia. In its early manifestations this is arterial, only becoming venous if long enough neglected. Congestion and pulp death follow in the natural course of events. Infection of the dead pulp is the usual result. As a rule, this takes place via the mouth, though it may be, and not infrequently is, of hematogenous origin. Unfortunately, this infection is not confined to the pulp itself, but passes through the apical foramen infecting the living tissues of the periapical region. Not infrequently this invasion is followed by acute symptoms—pain, swelling, temperature, etc., lasting for several days, or until the abcess which has formed has discharged itself, when it will subside into a chronic abcess with or without fistula, and usually without further local symptoms. More often there is local reaction of which the patient is unconscious, and this notwithstanding the fact that a chronic focus of infection exists, potentially more dangerous than the fistulous abcess. Reontgenographic examination of such teeth show varying conditions. In some instances there will be nothing to indicate that infection has taken place; the tissues about the root end of such teeth will have the appearance of normality. Negative findings however, do not preclude possible infection—a fact which should not be overlooked. Again, and this is the rule, certain changes will be observed to have taken place about the root apicis of such teeth. These may be represented by slight thickening of the periapical pericementum only, or by bone rarefaction extending all the way from the slightest radiolucency up to complete bone loss, often of considerable size. These areas are not necessarily abcesses and should not be so designated. They are however, indicative of infection and should receive serious consideration. Repeated bacteriological examinations show the strepticoccus viridans to be the usual infecting organism.

**Treatment.**

It is important in the treatment of non-vital teeth that the infection associated with them be eradicated as far as that is possible, without the loss of teeth. The indiscriminate extraction of teeth may be as unwise as the retention of those that are hopelessly infected. Extraction is of course one of the most efficient and expeditious ways of eliminating these infections and could it be done without impairment of the function of mastication, its practice might be justified. As a rule, however this cannot be done. Moreover, the esthetic must be considered. Loss of teeth make permanent changes in facial lines, a fact which should not stand in the way of the patient's health, but should nevertheless, receive consideration. Treatment of non-vital teeth consists in opening the tooth, gaining direct access to the pulp canals, and the removal of their septic contents. They must then be thoroughly sterilized and the pulp canal filled to its extreme end in such a way as to seal it to the apical foramen. This is not easily done and in not a few instances is impossible, as where roots are crooked or the pulp canal, more or less obliterated by secondary deposits. The first step in their treatment is good roentgenograms. Teeth shown to be seriously infected, or with roots that it is impossible to fill, should be extracted at once. Teeth that have been sterilized and opened to root end should again be X-rayed with wire markers in pulp canal to make sure that the apical end has been reached. Under most careful aseptic precautions, the root canal should then be filled (preferably with chlora-percha and gutta-percha points) and again X-rayed to ascertain whether the filling extends to the extreme end of root. This means that no one can successfully treat these cases whose office is not equipped with an X-ray outfit, inasmuch as it is imposible to *know* that they have been properly treated and filled without its use. Nor can dependence be placed upon the roentgenologist in the routine care of these cases; the work must be done by the person having the case under treatment if the best results are to be achieved. This should be borne in mind by the physician having such cases under his care. It may be said as a rule that teeth which cannot be sterilized and filled to the end of the root should be extracted. This should be made the invariable rule where focal infections are suspected of being the cause of the symptoms of which the patient complains. It may also be said that where teeth can be sterilized and filled to the end (and that usually includes all single rooted teeth) regeneration will take place and health be re-established in the tissues, except possibly in those relatively few cases where the apical pericementum has been destroyed. In this event, root amputation may be resorted to, and the tooth saved. In a word, non-vital teeth properly treated, can be preserved without prejudice to the general health. But, as indicated, "proper treatment" involves more than the ineffective treatment so often given these teeth. Where such treatment cannot be obtained extraction is indicated.

In cases, therefore, where focal infections are suspected about the teeth, the first thing should be a careful clinical examination by one qualified to make it. Second, a roentgenographic examination of all teeth not known to be vital. Third, based on these examinations, all teeth which it is believed impossible to save by treatment should be extracted. Fourth, all non-vital remaining, should then be opened, sterilized and their pulp canals filled to their apical foramen, each step of the operation being carefully checked up by roentgenographic examination. Only in this way can focal infections, associated with non-vital teeth, be eradicated and the possibility of metastatic infection from this source eliminated.

